# Longitudinal changes in infants' rhythmic arm movements during rattle-shaking play with mothers

**DOI:** 10.3389/fpsyg.2022.896319

**Published:** 2022-10-21

**Authors:** Zuzanna Laudanska, David López Pérez, Agata Kozioł, Alicja Radkowska, Karolina Babis, Anna Malinowska-Korczak, Przemysław Tomalski

**Affiliations:** Neurocognitive Development Lab, Institute of Psychology, Polish Academy of Sciences, Warsaw, Poland

**Keywords:** rhythm, motor development, infants, rhythmic arm movements, wearables, inertial motion units, wavelet coherence

## Abstract

From early on, infants produce a variety of rhythmic behaviors—an ability that likely supports later social communication. However, it is unclear, how this rhythmic motor production changes with age. Here, we investigated the coupling between infants' arm movements across the first year of life in a social context of a rattle-shaking play with their mothers. Through longitudinal measurements at 4, 6, 9, and 12 months of age using wearable motion trackers placed on infants' arms, we show that infants (*N* = 40) are similarly motivated to attempt rattle-shaking across the first year of life. However, with age, they make more rattling movements with an increased frequency. Their left and right arm movements become more coupled during rattle-shaking, as shown by an increase in wavelet coherence. Infants produced more rattling movements when they were rattling alone than when their mothers were rattling or singing simultaneously. There were no differences between infants' individual and social rattling in between-arms coherence. Our results may help to understand rhythmic arm movements as precursors of motor social coordination.

## 1. Introduction

Humans have the ability to produce rhythmic actions and coordinate their movements to external rhythms. Generally, a rhythm can be defined as a sequence of short and repeated intervals, with regularities that allow us to build expectancies when the next beat arrives (Jones, 1976), or as a recurrent non-random temporal pattern of actions that may not be strictly regular (Jaffe et al., 2001). Previous studies suggest that infants have the innate ability to process rhythms, since already newborns can detect on a cortical level the violation of the beat of a rhythmic sound sequence (Winkler et al., 2009) and the onset and offset of sound trains and changes in the presentation rate (Háden et al., 2015). In addition, it was shown that beat perception abilities are culture-specific (Hannon and Trehub, 2005). Furthermore, behavioral experiments demonstrated that 2-month-olds could discriminate between different musical rhythm patterns (Demany et al., 1977), even when the beat difference was small (Baruch and Drake, 1997), whereas 5-month-olds discriminated between different temporal groupings of audio stimuli (Chang and Trehub, 1977). Thus, it seems that rhythm perception and processing occurs from early on and become more specialized with age.

Overall, the majority of previous research was focused on children's perception and processing of rhythms. In contrast, the production of rhythmic actions by infants and children has been far less investigated. So far, it is known that across early development, infants produce various rhythmic behaviors (e.g., kicking, rocking, waving) with a peak period of rhythmic hand-banging around 6–7 months of age (Thelen, 1979, 1981). The ability to keep a steady beat and produce a spontaneous motor tempo emerges earlier than the ability to synchronize to an external beat (Provasi and Bobin-Bègue, 2003; Zentner and Eerola, 2010; Provasi et al., 2014). Infants' spontaneous motor tempo during drumming was observed from 5 months of age. It is slower than the adult one and it becomes faster and more regular with age (Rocha et al., 2021a,b). However, it is unknown whether the production of rhythmic movements changes during interactions with social partners.

The social context seems to modulate infants' production of some motor actions but only at a later age. For example, bouncing and rocking were displayed by 18-month-olds more often in the absence of a social partner (in a condition where they were presented with a non-social visual animation). In younger infants, at 10 months of age, these behaviors were not modulated by the presence or absence of a social partner (Rocha and Mareschal, 2017). A similar pattern was observed by Rocha and collaborators (Rocha et al., 2021a) during the drumming task—infants spent a higher proportion of time in rhythmic movement during the non-social trials. The social context seems to also facilitate joint drumming synchronization in preschool children (Kirschner and Tomasello, 2009; Yu and Myowa, 2021). Children's ability to coordinate their rhythmic activities with a partner develops between 18 and 30 months of age and studies with 18-month-olds have shown the crucial, facilitating role of a social partner's actions as opposed to those of a robot (Yu and Myowa, 2021).

Studying infants' rhythmic actions in the context of social interactions could be key to better understand whether these rhythmic actions form a foundation for later social communication (Jaffe et al., 2001). Infants early on start to engage in proto-conversations with their caregivers, in which infants' movement patterns are very responsive to the time structure of their mothers' movements (Trevarthen, 1979). Communication with others and verbal dialogues are also rhythmic activities, where both timing and synchronization of own actions with the interlocutor's actions are crucial (Jaffe et al., 2001). Production of rhythmic movements—especially the ones that result in multimodal feedback such as drumming or rattling—may be an opportunity for infants to learn about contingencies between their actions and outcomes of those actions. Repetitive and recurrent movements are also an opportunity to practice specific types of limb movements and master their execution. Since motor coordination is another important aspect of dyadic interactions, it seems key to better understand its early precursors during rhythmic actions. Altogether, more studies are needed to describe the role of a social partner in rhythmic activities and how these rhythmic actions may form the foundation for later social communication (Jaffe et al., 2001).

In the present study, we investigated how infants' spontaneous rhythmic behavior in the social context of play changes in development. Our main goal was to study the developmental changes in motor coordination between arm movements during rattle-shaking. Furthermore, we also studied whether infants produce more rhythmic arm movements as they grow older and whether they do it at a higher frequency. Additionally, we explored the role of the social partner in infants' rattling. We studied the changes in rhythmic arm movements in a naturalistic set-up: mother-infant dyads were invited to play together in the lab. Their interactions were video-recorded, which enabled us to annotate, during which episodes infants were rattling alone and during which mothers were rattling or singing alongside each other. We have compared these categories in exploratory analyses to see whether there are potential differences between infants' individual and social rattling.

To this end, we first recorded infants' arm movements using wearable motion trackers (Inertial Motion Units, IMUs) in a rattle-shaking task during parent-infant interactions when infants were around 4, 6, 9 and 12 months of age. Secondly, we identified and manually annotated the episodes when infants were rattling to include only this type of activity in further analyses. Thirdly, we classified episodes of infant rattling into two categories: “Mother Not Providing Rhythm” in instances where the infant was rattling alone and “Mother Providing Rhythm” in instances where during the infant's rattling, the parent was providing them with auditory stimulation by rattling or singing. Fourthly, we calculated the number of rattle-shakes (i.e., infant arm movements with a rattle) in a data-driven way. This, in turn, allowed us to calculate the rattling frequency and the coordination between the movements of both arms. To assess the degree of coordination between the infants' two arms, we used wavelet coherence, which captures information on a range of constituent frequencies of the signals across the recorded interaction (e.g., Grinsted et al., 2004; Hale et al., 2020).

We hypothesized that (1) infants would be able to produce more rhythmic arm movements with age (Rocha et al., 2021a,b), (2) they would rattle at a higher frequency with age, and (3) their between-arms coordination (measured with wavelet coherence) would increase with age. The analyses regarding the effect of rattling alone vs. rattling with a mother were exploratory and we did not have any a priori hypotheses.

## 2. Methods

### 2.1. Participants

Participants were 40 Polish mother-infant dyads from an ongoing longitudinal study on infant limb movement during social interactions and language development. Participants were invited to the lab when the infants were around 4 (T1), 6 (T2), 9 (T3), and 12 (T4) months old. Six infants contributed data at all four time points, whereas 34 infants missed one visit (mostly due to Covid-19 related restrictions). Therefore, 20 infants contributed data at T1, T2 and T3, 9 at T2, T3, and T4, and 5 at T1, T3 and T4 (see Table 1 for an overview of sample characteristics). Participants were from predominantly middle-class families living in the Warsaw metropolitan area. The majority of mothers had completed higher education: 35 held a master's degree, 2 held a bachelor's and 2 completed high school (1 missing data). For their participation, infants received a diploma and a small gift (a baby book). The study received clearance from the Ethics Committee at the Institute of Psychology, Polish Academy of Sciences.

**Table 1:** Sample characteristics.

**Time point**	***N***	**Number of girls**	**Number of boys**	**Mean age in months (SD)**	**Min age in months**	**Max age in months**
T1	31	10	21	4.35 (0.29)	3.90	5.20
T2	35	13	22	6.55 (0.36)	6.00	7.40
T3	39	13	26	9.14 (0.39)	8.60	10.20
T4	21	4	17	12.05 (0.37)	11.60	13.10

### 2.2. Procedure

**Figure 1:**
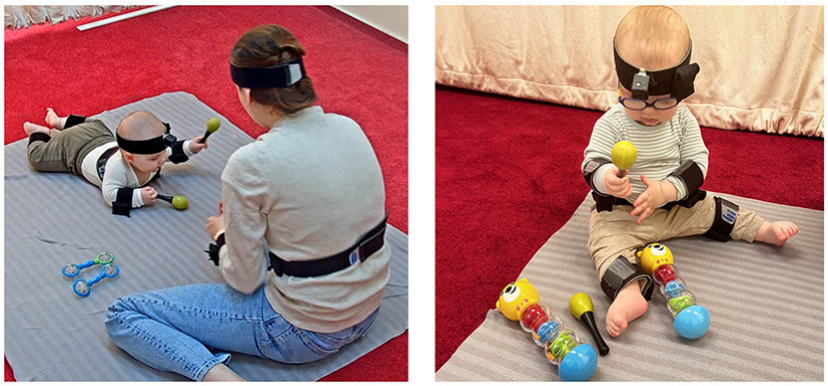
Photos of the toys used in the rattle-shaking play at T1 and T2 **(left)**, T3 and T4 **(right)**. Signed permission of the caregiver was acquired for the publication of the images.

Infant-parent interactions were recorded in a laboratory room, in a carpeted play area. Upon the family's arrival, an experimenter explained the study protocol and obtained parental written consent. Once the infant was familiarized with the laboratory, the wearable motion trackers attached to elastic bands were put on the infants' and caregivers' bodies. Then, the infant-parent dyads took part in a series of interactive games with different sets of age-appropriate toys. There were 6–7 different tasks during each meeting, but here we report data only from the rattle-shaking task. In this task, which lasted approximately 5 min, the caregivers were instructed to play with their infants using the provided rattles in their preferred way. They were given two maracas rattles and two other rattles of different types (smaller and lighter barbell rattles at T1 and T2 and bigger teddybear rattles at T3 and T4, see Figure 1). At the beginning of each game, the caregivers were asked to clap several times to mark the start of the procedure to synchronize wearable sensors with video recordings. The infants' body position was not constrained and both the mother and the infant were free to move around the room. Therefore, the sitting arrangement varied between visits and could change during each visit. The most common body position at T3 and T4 was independent sitting, whereas for T1 and T2 was lying either in a prone or a supine position.

### 2.3. Equipment

Infants' and mothers' body movements were recorded at 60 Hz using wearable motion trackers (MTw Awinda, Xsens Technologies B.V.) connected wirelessly through an Awinda station receiver (Xsens Technologies B.V.) and synchronized in real-time with MT Manager Software (Xsens Technologies B.V.). Overall, 12 sensors were used (on the infant's arms, legs, head, and torso, see Figure 1; and on the caregiver's arms, head, and torso), but in this paper, we report data only from two sensors placed on infant's arms.

The interactions were recorded with three remote-controlled CCTV color cameras in HD quality. During the interaction, an experimenter operated the cameras (this included zooming in and out as well as moving them vertically and horizontally) to ensure that at least one camera captured the infant's behavior.

### 2.4. Manual annotation of rattling

In each video recording, the episodes when infants were rattling as well as mothers clapping for the purpose of synchronization of videos with wearable data were manually annotated by a trained coder in ELAN 6.3 (Sloetjes and Wittenburg, 2008; ELAN 6.3, 2022). Firstly, the onset and offset of each clap were identified in a frame-by-frame manner to precisely include the moment of acceleration before joining hands. Secondly, the onset and offset of each infant rattling episode were annotated. We defined a rattling episode as a period when an infant was holding at least one rattle and made at least one movement that produced the rattling noise. Instances of an infant generating the rattling sound unintentionally (e.g., while holding a rattle during crawling or throwing it) were not annotated. Each episode ended if (1) the infant dropped the rattle or (2) was holding the rattle but not making any arm movements. Periods when an infant did not wear motion trackers on both arms were not annotated and excluded from the analyses. Periods when the mother was moving infant's arms were not annotated. In the second pass of coding, each rattling episode was assigned to a category: either (1) Mother Not Providing Rhythm (infant's rattling alone while the mother was not providing an auditory rhythm) or (2) Mother Providing Rhythm (this included rattling at the same time as the mother was rattling, singing, or both simultaneously, and rattling directly after the mother had finished rattling or singing).

In total, 126 videos were annotated. Videos during which mothers did not clap were excluded from further analyses (*N* = 3, two at T1, one at T3) due to problems with synchronizing motion trackers' data with video recording. Similarly, videos during which the infant did not make any rattling movements were excluded from further analyses (*N* = 5, two at T1, two at T2, one at T3). In order to establish the inter-rater reliability, 26 randomly selected videos (20%) were annotated separately by two trained coders. Inter-rater reliability was performed in ELAN and estimated using Cohen's κ statistic, which takes into account chance agreement. The mean Cohen's κ for rattling episodes was 0.79, which can be interpreted as substantial agreement (Landis and Koch, 1977).

### 2.5. Data pre-processing

Acceleration data from sensors placed on both wrists of an infant were processed in Matlab (Mathworks, Inc., Natick, USA) using in-house scripts. First, missing samples were identified and interpolated using the *interp1* function with cubic spline interpolation of the values at neighboring grid points. Then we collapsed the kinematic vectors obtained from the IMUs into a unique normalized dimension (a one-dimensional overall acceleration time series) as follows:


(1) $$\begin{array}{l} Acc=\sqrt{x{\left(t\right)}^{2}+y{\left(t\right)}^{2}+z{\left(t\right)}^{2}} \end{array}$$

where *Acc* is the normalized acceleration


(2) $$\begin{array}{l} x,y,z\in {\mathbb{R}}^{1\times N} \end{array}$$

and *a*_*x*_, *a*_*y*_, and *a*_*z*_ are the kinematic acceleration vectors in x, y, and z dimensions respectively at each time point *t*. Next, data were smoothed using the *medfilt1* function that applies a third-order median filter to remove one-point outliers by replacing each value with the median of three neighboring entries (see Supplementary Figure 1 for an example of the sensor time series).

### 2.6. Synchronization of sensor data and annotated videos

Video and sensor data for each infant and visit were later synchronized using the mothers' clapping (see Supplementary Section 2 for an example). To this end, a graphical user interface (GUI) loaded the sensor data to manually select the period when the clapping occurred (Supplementary Figures 1, 2). Then, we categorized the manually selected sensor periods from the GUI as “1” and “0”, where 1 indicated movements that were one standard deviation above the mean acceleration in that period and 0 otherwise. Next, the time series outside the selected clapping period was set to 0. Finally, we merged those automatically detected claps separated by 50 ms or less to avoid artifactual claps due to extremely short claps or claps close together. This process resulted in a time series that contained only the mothers' claps. In the next step, this was used to find the delay between the IMUs data and the manually coded video data. To find this delay, we used diagonal cross-recurrence quantification analysis (DCRP) (e.g., Richardson and Dale, 2005) using two different time windows (a shorter window of 6 s and a longer one of 15 s). We calculated the lag profile using a Matlab version of the R function *drpdfromts* (CRQA R-package) (Coco and Dale, 2014). Generally, the experimenter initiated video and sensor recordings closely in time, so the lag between them usually was not longer than 6 s. Initially, the algorithm estimated the delay using the 6 s time window and loaded a GUI plotting both the sensor data and the manually coded data (see Supplementary Figure 3). This process asked the user to visually inspect and validate the proper alignment of the data. In 7% of cases, the lag between sensor and video data was longer than 6 s. Therefore, in these cases, we repeated the previous step, using a 15 s-long time window. Again, the alignment was visually inspected. Further analyses were performed on the temporally aligned time series.

### 2.7. Wavelet coherence analysis of arm movements

Wavelet coherence (WC) is a relative measure of how well-correlated the power and phase of two signals are at a given frequency and time (Grinsted et al., 2004) and it is defined as the squared absolute value of the smoothed cross-wavelet spectrum normalized by the product of the smoothed individual wavelet power spectra, as follows:


(3) $$\begin{array}{l} WC=\frac{|S\left(C_{x}^{*}\left(a,b\right)C_{y}\left(a,b\right)\right){|}^{2}}{S\left(|C_{x}\left(a,b\right){|}^{2}\right)\cdot S\left(|C_{y}\left(a,b\right){|}^{2}\right)} \end{array}$$

where *Cx(a,b)* and *Cy(a,b)* denote the continuous wavelet transforms of *x* and *y* (with x and y indicating time series of an infant's left and right arm movements) at scales *a* in frequency and positions *b* in time. The superscript * is the complex conjugate and *S* is a smoothing operator in time and scale. The dot in the denominator indicates a product between the individual wavelet spectra of both time series. Wavelet coherence has a value between 0 and 1, where 0 means that no coherence is present between signals and 1 means that both signals are fully coherent at any given time and frequency. Wavelet coherence closely resembles a traditional correlation coefficient, and it can be interpreted as a localized correlation coefficient in time-frequency space (Grinsted et al., 2004).

**Figure 2:**
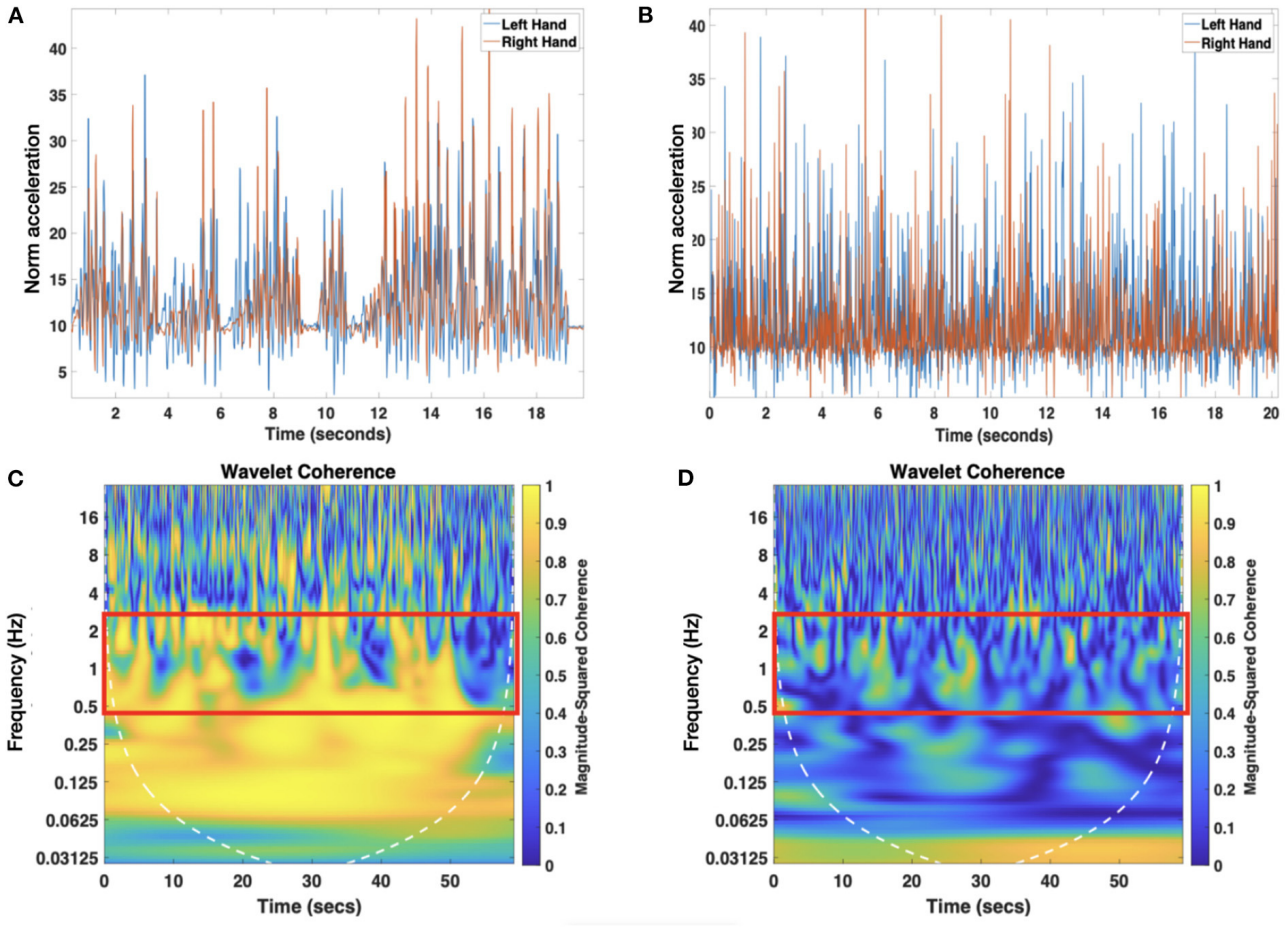
Example of the time series created by joining together the rattling episodes of both arms using the manually annotated data **(A)**. Panel **(B)** represents the randomized version of the rattling time series. Only the first 20 s are shown to ease representation. Panels **(C,D)** represent the wavelet coherence spectra of movements of both arms using the original rattling time series and the randomized version, respectively. Highlighted with a red rectangle are the areas where the average wavelet coherence was computed.

Here, we estimated the wavelet coherence between movements of both hands using the *wcoherence* function in Matlab. To this end, manually annotated episodes of rattling were used to estimate the average duration of each rattling episode and to segment the wearable data (see Figure 2A for an example and Figure 2C for its computed wavelet coherence spectra) and to identify the number of rattling movements using an in-house Matlab script. We estimated rattling movement events following the same approach we used to calculate the clapping events. We categorized the rattling periods as “1” and “0”, where 1 indicated movements that were one standard deviation above the mean acceleration and 0 otherwise. Then we merged those automatically detected movements separated by 50ms or less to avoid artifactual rattling events due to extremely short movements or movements close together. Next, the rattling frequency was calculated as the number of rattling movements divided by the total duration of rattling time derived from the video annotation data (see Table 2 for descriptives). In all but two visits, infant rattling was within the range of 0.5 and 2.5 Hz. Two visits (one at T2 and one at T4) that had the rattling frequency above 2.5 Hz were considered outliers and excluded from the analysis. Given the range of rattling frequencies (see (2) for descriptives), we calculated the average wavelet coherence coefficient within the range of 0.5 and 2.5 Hz for each visit.

**Table 2:** Descriptive statistics at each time point.

	**T1**			**T2**			**T3**			**T4**		
	**Mean (SD)**	**Min**	**Max**	**Mean (SD)**	**Min**	**Max**	**Mean (SD)**	**Min**	**Max**	**Mean (SD)**	**Min**	**Max**
Number of rattling episodes during play	10.65 (7.16)	3.00	27.00	12.66 (8.39)	2.00	30.00	15.57 (8.31)	2.00	33.00	16.90 (7.11)	4.00	30.00
Mean duration of rattling episode [s]	1.51 (0.85)	0.46	4.20	2.59 (1.14)	0.81	6.03	3.08 (1.25)	0.94	6.14	2.90 (1.30)	1.33	5.59
Number of rattling movements during play	17.77 (10.75)	2.00	39.00	38.69 (33.10)	5.00	141.00	62.54 (31.48)	6.00	124.00	83.60 (45.08)	17.00	195.00
Rattling frequency [Hz]	1.09 (0.38)	0.49	2.43	1.10 (0.28)	0.55	1.72	1.22 (0.31)	0.62	1.93	1.43 (0.34)	0.95	2.18
Wavelet coherence	0.38 (0.12)	0.21	0.73	0.31 (0.10)	0.12	0.58	0.42 (0.13)	0.19	0.79	0.55 (0.15)	0.30	0.78

Finally, we conducted a control analysis by calculating wavelet coherence between the right and the left arm on the shuffled time-series data from each participant and comparing the mean coherence values of the shuffled data with the original data from all participants. The procedure was iterated 1000 times. This allowed us to show that the wavelet coherence between hand movements did not arise randomly (see Figure 2B for an example of the randomized time series and Figure 2D for its wavelet coherence spectra).

In addition, to investigate developmental changes in movements of a single hand we calculated the continuous wavelet transform spectra (see Supplementary Information 2).

### 2.8. Statistical analysis

**Figure 3:**
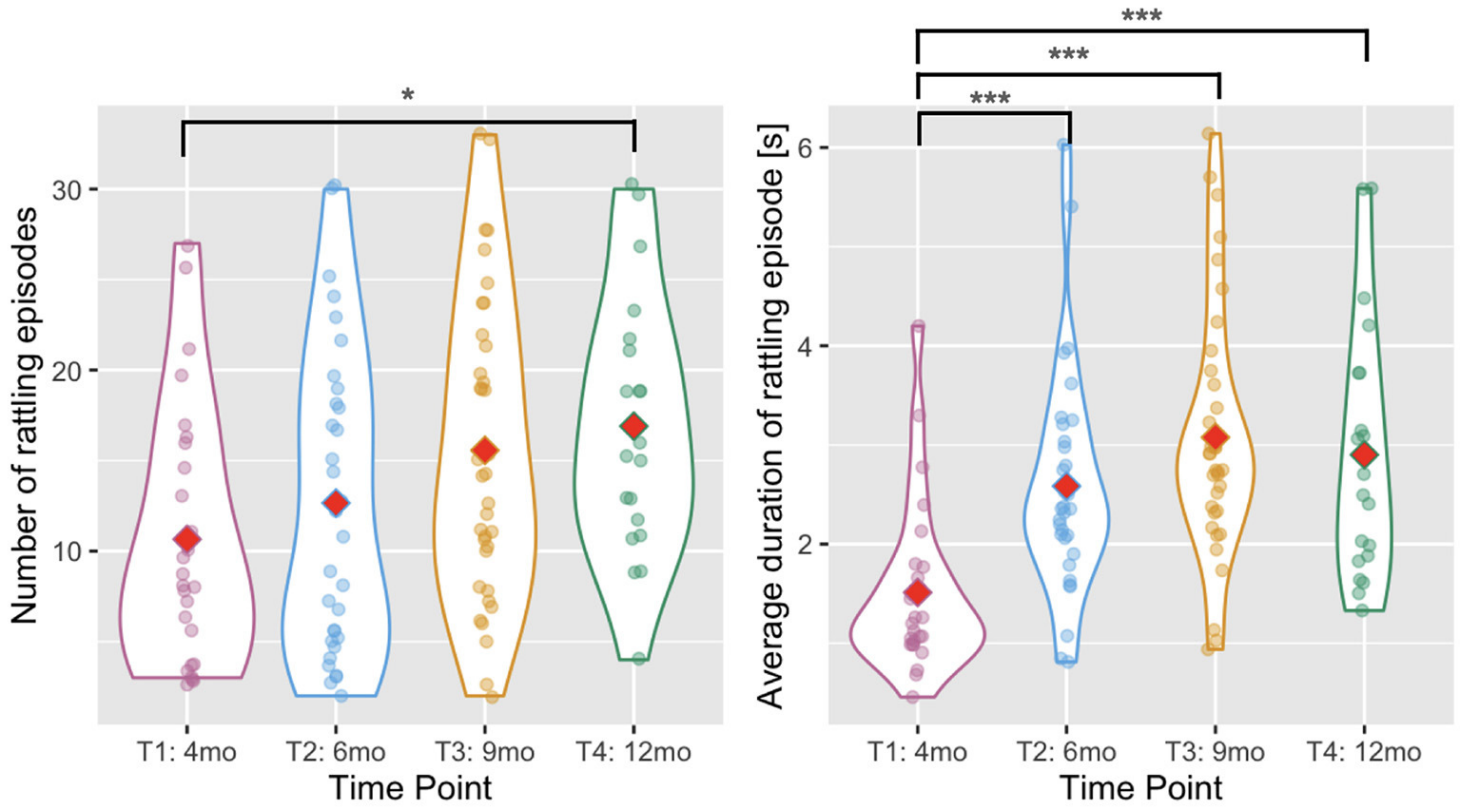
Violin plots showing the number of rattling episodes **(left)** and the average duration of rattling episode **(right)** across time points. Red diamonds indicate mean values. A single asterisk indicates significance at *p* < 0.05, two asterisks indicate *p* < 0.01, and three indicate *p* < 0.001.

**Figure 4:**
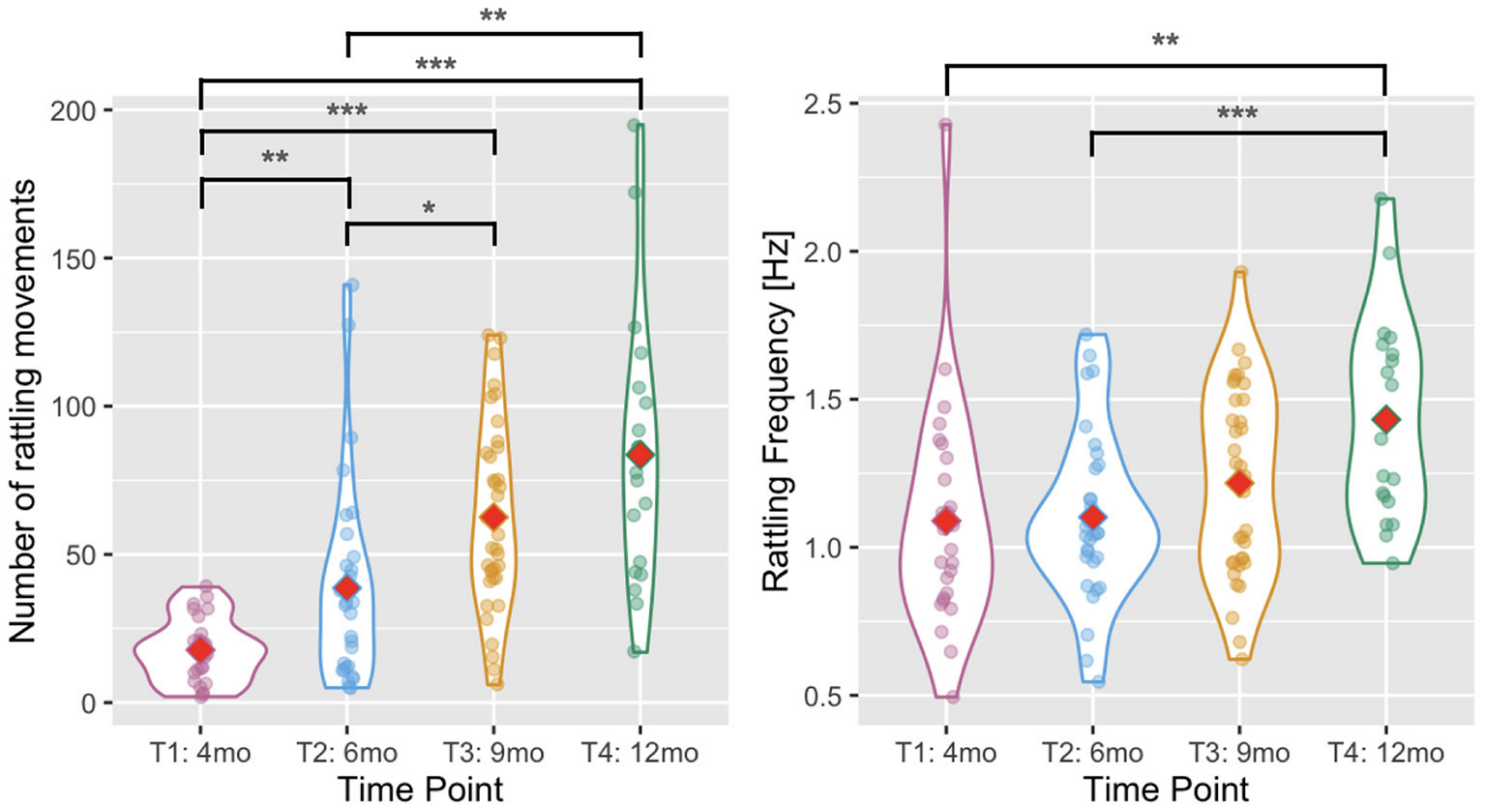
Violin plots showing the number of rattling movements **(left)** and the rattling frequency **(right)** across time points. Red diamonds indicate mean values. A single asterisk indicates significance at *p* < 0.05, two asterisks indicate *p* < 0.01, and three indicate *p* < 0.001.

**Figure 5:**
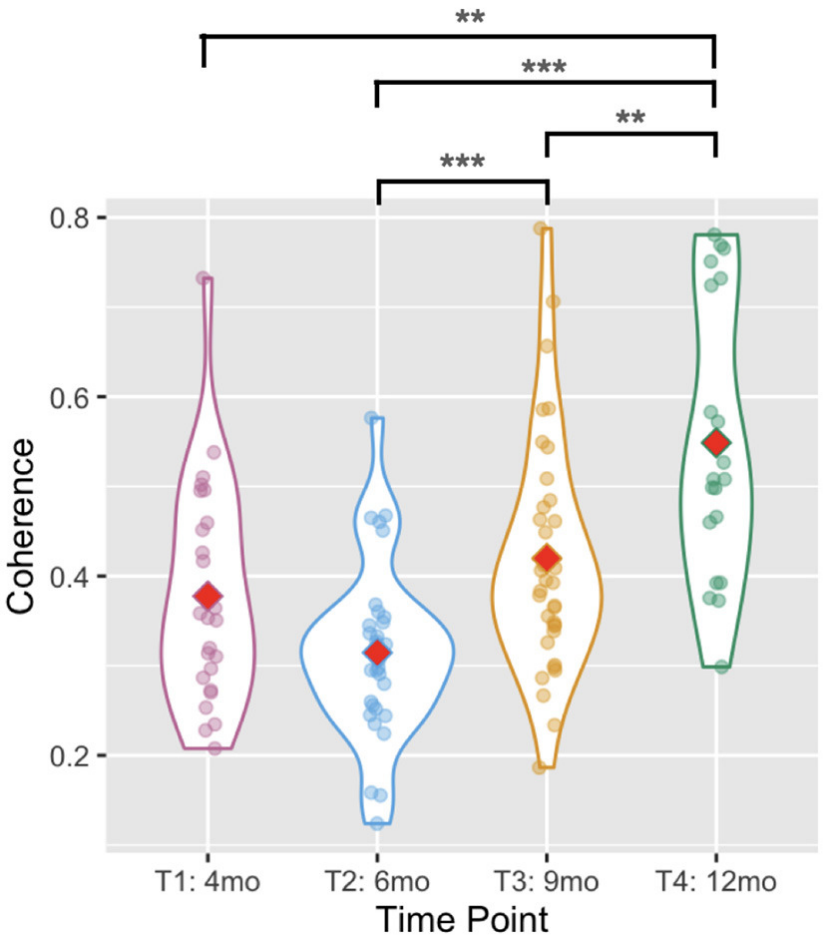
Violin plots showing the between-arms coherence. Red diamonds indicate mean values. Two asterisks indicate *p* < 0.01, and three indicate *p* < 0.001.

**Figure 6:**
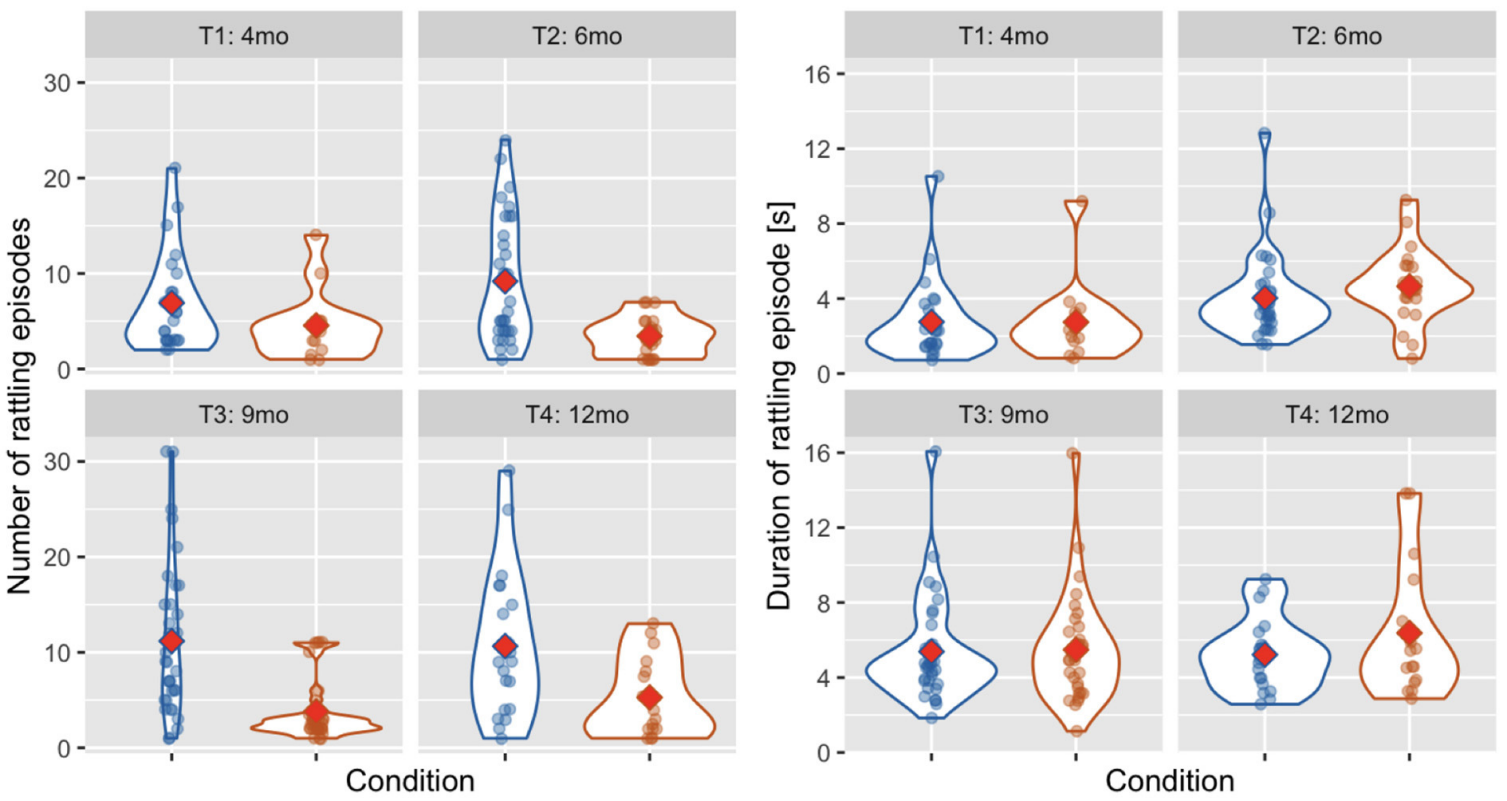
Violin plots showing the number of rattling episodes **(left)** and the average duration of rattling episode **(right)** across time points and conditions: mother Not Providing Rhythm (blue) and Mother Providing Rhythm (orange). Red diamonds indicate mean values.

**Figure 7:**
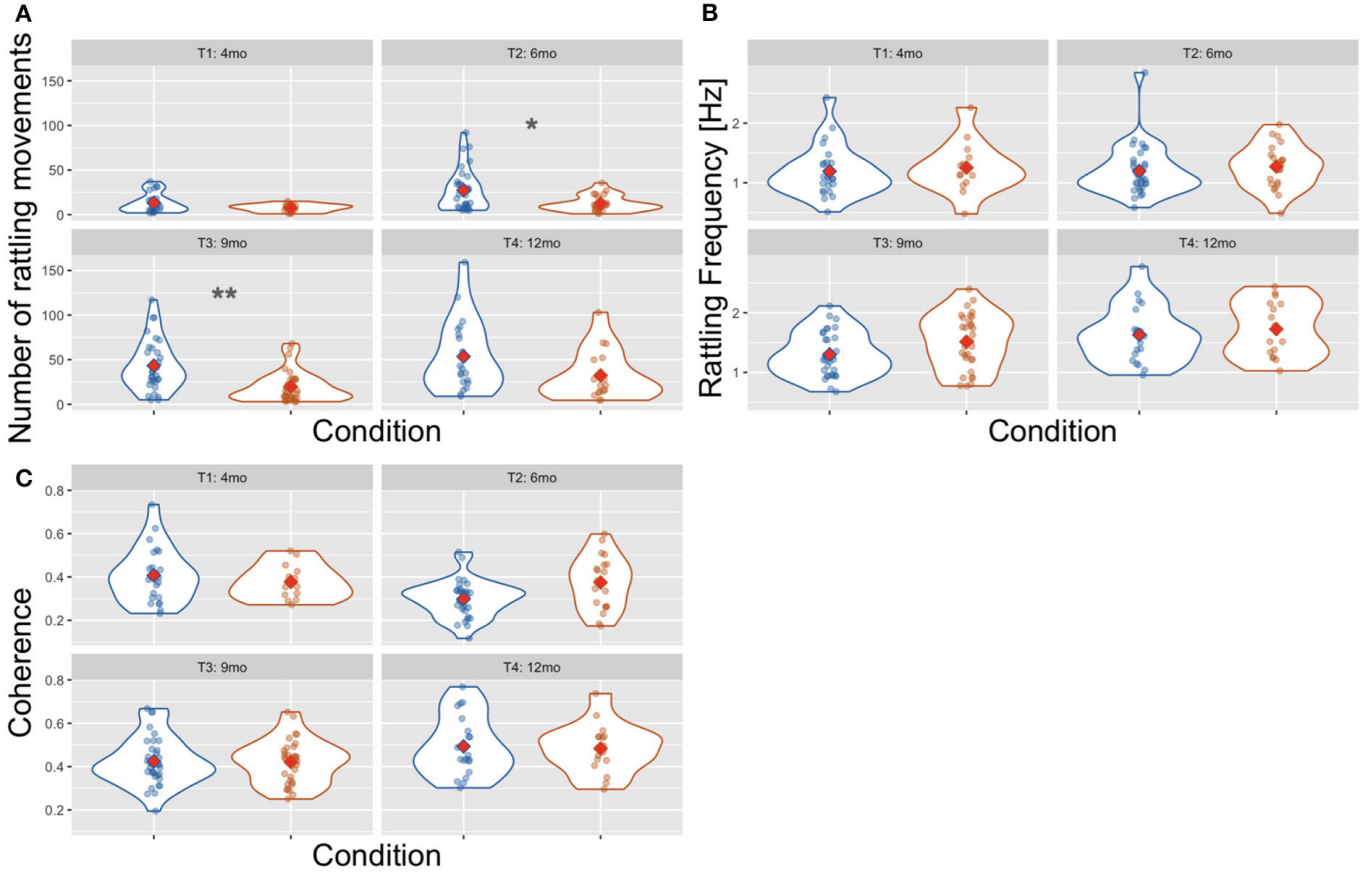
Violin plot showing the number of rattling movements **(A)**, the frequency of rattling **(B)**, and the between-arms coherence **(C)** across time points and conditions: Mother Not Providing Rhythm (blue) and Mother Providing Rhythm (orange). Red diamonds indicate mean values, a single asterisk indicates significance at *p* < 0.05, and two asterisks indicate *p* < 0.01.

First, to investigate the developmental changes in the number of rattling episodes, their mean duration, the number of rattling movements, the frequency of rattling and the between-hands coherence we ran General Estimating Equations (GEEs) with a Bonferroni correction for pairwise comparisons with age as a repeated measure (T1, T2, T3, T4). Second, to explore potential differences between infants' spontaneous and social rattling, we ran GEEs with age (T1, T2, T3, T4) and condition (Mother Not Providing Rhythm vs. Mother Providing Rhythm) as repeated measures. GEEs are particularly adequate for longitudinal data because they take into account the dependency and ordering of the data within subjects in repeated-measures designs. Data analysis was conducted in IBM SPSS Statistics 26, Figures 1–7 were created using (R Core Team, 2020) and RStudio, version 1.4.1106 (RStudio Team, 2020), and ggplot2 package (Wickham, 2016).

Finally, for control purposes, we run two control analyses. In the first one, we excluded infants who had the lowest numbers of rattling episodes (7 rattling episodes or less) to see whether the infrequent rattlers affected the pattern of results. The significance of all main effects remained unchanged apart from the effect of age on the number of rattling episodes (see Supplementary Information 1 for the full overview). In the second one, we have re-coded our video data to include only those rattling episodes during which infants consecutively performed at least 4 arm movements in a row that produced a rattling sound. Again, the significance of main effects remained unchanged apart from the effect of age on the average duration of a rattling episode (see Supplementary Information 3 for the full overview).

## 3. Results

### 3.1. Number of rattling episodes and the average duration of an episode

The number of rattling episodes (annotated periods when an infant was holding at least one rattle and made at least one movement that produced rattling noise) slightly increased with age [Wald χ^2^(3)= 10.448, *p* = 0.015, see Figure 3 and Table 2 for descriptive statistics] as the number of episodes increased between T1 and T4 (*p* = 0.026). There was also a main effect of age in the analysis of the average duration of a rattling episode [Wald χ^2^(3) = 38.450, *p* < 0.001, see Figure 3]. The duration was shorter at T1 than at T2, T3, and T4 (all *p*s < 0.001).

### 3.2. Number of rattling movements

We predicted that infants would be able to produce more rhythmic arm movements with age. To test this hypothesis, we took the number of rattling movements detected automatically in the movement time series during annotated rattling episodes. The number of rattling movements increased with infants' age [Wald χ^2^(3) = 129.804, *p* < 0.001, see Figure 4], and pairwise comparisons showed that there were significantly fewer rattling movements at T1 than at T2 (*p* = 0.002), T3 (*p* < 0.001), and T4 (*p* < 0.001); and fewer at T2 than at T3 (*p* = 0.018) and T4 (*p* = 0.001). The difference between T3 and T4 was not significant (*p* = 0.465).

### 3.3. Rattling frequency

The rattling frequency (i.e., number of rattling movements divided by the total duration of rattling time) increased with infants' age [Wald χ^2^(3) = 20.498, *p* < 0.001, see Figure 4] and it was higher at T4 than at T1 (*p* = 0.007) and T2 (*p* < 0.001). The difference between T4 and T3 did not reach significance (*p* = 0.058).

### 3.4. Between-arms coherence

Average wavelet coherence increased with age [Wald χ^2^(3) = 49.795, *p* < 0.001, see Figure 5] between T2 and T3 (*p* < 0.001) and between T3 and T4 (*p* = 0.009). It was higher at T4 than at T1 (*p* = 0.001) or T2 (*p* < 0.001). The difference between T1 and T2 was not significant (*p* = 0.224), similarly there was no difference between T1 and T3 (*p* = 0.725).

### 3.5. Mother providing vs. not providing rhythm

To explore whether there are any differences in the rhythmic movements that infants produce on their own without an external beat (Mother Not Providing Rhythm) and movements that they produce while being accompanied by their mother through rattling or singing (Mother Providing Rhythm), we have compared these two categories in additional analyses.

#### 3.5.1. Number of rattling episodes and the average duration of an episode across categories

Overall, 69.44% of rattling episodes were classified as Mother Not Providing Rhythm and 30.55% as Mother Providing Rhythm. This proportion was similar across time points (Mother Not Providing Rhythm at T1: 71.03%, T2: 73.02%, T3: 68.92%, T4: 64.53%). Difference in the number of rattling episodes between categories was statistically significant [Wald χ^2^(1) = 30.249, *p* < 0.001, see Figure 6]. There was no main effect of age [Wald χ^2^(3) = 4.893, *p* = 0.180] and the interaction between age and condition was also not significant [Wald χ^2^(3) = 6.854, *p* = 0.077].

In the average duration of a rattling episode (see Figure 6), there was no effect of condition [Wald χ^2^(1) = 2.072, *p* = 0.150] and the age × condition interaction was also not significant [Wald χ^2^(3) = 2.077, *p* = 0.556]. There was, however, a main effect of age [Wald χ^2^(3) = 25.112, *p* < 0.001], as the average duration of a rattling episode was shorter at T1 than at T2 (*p* = 0.033), T3 (*p* < 0.001), and T4 (*p* = 0.001).

#### 3.5.2. Number of rattling movements across categories

There were more rattling movements in the Mother Not Providing Rhythm category than in the Mother Providing Rhythm category [Wald χ^2^(1) = 17.880, *p* < 0.001, see Figure 7]. The number of rattling movements increased with infants' age [Wald χ^2^(3) = 100.117, *p* < 0.001] as it was higher at T4 than at T1 (*p* < 0.001) and T2 (*p* = 0.001); and higher at T3 than at T1 (*p* < 0.001) and T2 (*p* = 0.012), and higher at T2 than at T1 (*p* < 0.001). There was also a significant interaction effect of age × condition [Wald χ^2^(3) = 13.548, *p* = 0.004] as there were more rattling movements in the Mother Not Providing Rhythm condition than in the Mother Providing Rhythm condition at T2 (*p* = 0.013) and T3 (*p* = 0.003).

#### 3.5.3. Rattling frequency across categories

In the rattling frequency (see Figure 7), there was no effect of condition [Wald χ^2^(1) = 3.578, *p* = 0.059] and the age × condition interaction was not significant [Wald χ^2^(3) = 1.860, *p* = 0.602]. There was a main effect of age [Wald χ^2^(3) = 25.168, *p* < 0.001] as the rattling frequency was higher at T4 than at T1 (*p* < 0.001), T2 (*p* < 0.001), and T3 (*p* = 0.021).

#### 3.5.4. Between-arms coherence across categories

There was no difference in the average between-arms coherence across conditions [Wald χ^2^(1) = 0.608, *p* = 0.435, see Figure 7] and the interaction of age × condition was also not significant [Wald χ^2^(3) = 6.436, *p* = 0.092]. The average coherence increased with age [Wald χ^2^(3) = 38.427, *p* < 0.001] as it was higher at T4 than at T1 (*p* = 0.014) and T2 (*p* < 0.001); and higher at T3 than at T2 (*p* < 0.001).

### 3.6. Control comparisons with shuffled time series

To show that the wavelet coherence of between-arm movements did not arise randomly, we conducted a control analysis by calculating wavelet coherence between the right and the left arm on the shuffled time-series data from each participant and comparing the mean coherence values of the shuffled data with the original data from all participants. These comparisons showed that at T2 coordination between both arms is not different from noise [T2: *t*_(32)_ = 0.043; *p* = 0.966]. For T1, T3, and T4 the coherence for observed data was significantly higher than their corresponding shuffled data [T1: *t*_(25)_ = 2.555; *p* = 0.017; T3: *t*_(36)_ = 5.800; *p* < 0.001; T4: *t*_(20)_ = 6.904; *p* < 0.001]. The difference between observed and shuffled data at T1 was not significant in the control analysis with excluded infrequent rattlers (see Supplementary Analysis 1).

## 4. Discussion

Here we investigated how infants' spontaneous rhythmic behavior in the social context of play with rattles changes across the first year of life. Through precise longitudinal measurements using wearable motion trackers, we show that infants are highly motivated to produce rhythmic manual actions that generate multimodal feedback (rattle-shaking). The number of rattling episodes (periods when the infant was holding at least one rattle and made at least one movement that produced rattling noise) is similar across all visits, suggesting that infants are similarly motivated to attempt rattle-shaking. The mean duration of rattling episodes increases in subsequent months in comparison to the first visit at 4 months as infants' motor control and grasp strength increase. As infants grow older, they also make more rattling movements and their frequency of rattling increases. Furthermore, infants' arm movements become more coupled during rattle-shaking, as shown by the age-related increase in wavelet coherence - and this effect was consistent across control analyses with stricter inclusion criteria. In an additional analysis we also observed the developmental increase in power of wavelet spectra of movements of a single hand, with power being highest in the frequency range between 2 and 3 Hz consistently at all time points. This suggests that across the first year of life it is not the frequency of rattling that changes, but the organization of rhythmicity within the same frequency range. Finally, infants also produced more rattling movements (at the age of 6 and 9 months) when they were rattling alone compared to when mothers provided them with auditory stimulation of rattling or singing. This effect could reflect the caregivers approach to give their infants time for individual exploration of a new interesting objects (rattles) in a novel situation (laboratory). There were no differences between infants' rattling alone and infants' rattling with their mothers in the average between-arms coherence, rattling frequency, or average duration of rattling episode.

In our study, we investigated motor aspects of infants' rattle-shaking in the context of interactions with their mothers. Overall, our results suggest a developmental increase in arm movements during rattle-shaking play with a mother. Younger infants, at 4 and 6 months of age, seem to make fewer rhythmic arm movements which could be explained by their immature motor control (Goldfield, 1995). Motor control at the subcortical level of the central nervous system emerges and matures mainly during the first year of life, allowing for essential trunk stabilization and body positioning, a prerequisite for reaching and grasping arm movements (Westcott et al., 1997; Dusing and Harbourne, 2010; Kobesova and Kolar, 2014), both of which are necessary for the execution of rhythmic rattling. With emerging postural control, arms can also be less involved in stabilizing the body posture and used more in skilled manual reaching (Hadders-Algra, 2005). Our finding of an increase in the frequency of rattling in the second half of the first year of life suggests that older infants can execute rattling movements with more ease. This is in line with a previous study, which recently showed that infants' movements during drumming become faster and more regular with age (Rocha et al., 2021a). We also observed a developmental increase in the infants' between-arms coherence, which shows that arm movements become more coupled during rattle-shaking across the first year of life. On the one hand, this could be explained by the fact that older infants are able to play comfortably in a position and do not need one hand to support themselves while sitting or lying in a prone position. On the other hand, this could be related to an increase in the overall spontaneous rhythmicity of movement. As Hoehl et al. (2021) stated in their review, rhythmic synchronization is usually not limited to a movement of a single limb, but it diffuses throughout the body.

All in all, our results shed more light on the development of the infants' spontaneous rhythmic actions during play with the caregiver. We show that infants are motivated to play with rattles already at 4 months and they keep trying to produce rhythmic arm movements despite constraints related to their limited strength and ability to stay comfortably in a given body position. The number of rattling movements that they produce and the frequency of those movements increase across the first year of life. Similarly, the level of their between-arms coordination also increases with age.

Our findings on spontaneous rhythmic actions during early interactions are highly relevant to the understanding of early underpinnings of conversational skills. First, the motivation to keep producing spontaneous rhythmic movements that result in multimodal feedback may be beneficial for learning about the structure of early social interactions and proto-conversations. Interpersonal communication shows many rhythmic properties. Rhythmic patterns provide information necessary to predict and anticipate the other person's actions (Warner, 1992). Effective communication requires reciprocity, being responsive to the interlocutor (Sebanz et al., 2006) and becoming in-sync on many different levels (e.g., Feldman, 2007; Feldman et al., 2011; Dumas and Fairhurst, 2021). It also requires an understanding of timing to be able to participate in vocal turn-taking (e.g., Gratier et al., 2015). Second, infants' rhythmic movements are considered by parents as communicative signals. Caregivers respond to them frequently, especially when they co-occur with infants' signals from other modalities (vocalizations or gaze toward parents; Moreno-Núñez et al., 2021). Third, rhythmic arm movements are postulated to be the precursor of vocal-entangled gestures that accompany day-to-day adult communication (Pouw and Fuchs, 2022). As was shown in previous studies (Thelen, 1979; Locke et al., 1995; Ejiri, 1998; Ejiri and Masataka, 2001; Iverson and Fagan, 2004; Iverson and Wozniak, 2007; Burkhardt-Reed et al., 2021), rhythmic manual movements often co-occur with infants' vocalizations and this co-occurrence is observed at much earlier developmental stages than other types of gestures—such as pointing (emerging around 12 months of age; Colonnesi et al., 2010; Murillo et al., 2021) or iconic gestures (emerging around 26 months of age, Ozcaliskan and Goldin-Meadow, 2011). Thus, it seems that rhythmic arm movements that accompany vocal learning may serve as a precursor to the gesture-speech system (Iverson and Fagan, 2004; Pouw and Fuchs, 2022).

As rattling is a multimodal signal, it creates a unique opportunity to practice rhythmic arm movements and motor control with encouragement from the parent's side. Caregivers can facilitate infants' rattling by handing in the rattles but also by rattling in-sync or producing an external beat that infants can entrain to (singing or reciting nursery rhymes). This allows infants to practice coordinating their movements with the movements produced by the caregiver, yet these aspects of motor social coordination have been scarcely investigated in infancy (e.g., Scola et al., 2015). Overall, improvements in spontaneous rhythmic production, especially one that provides auditory feedback (such as rattling and drumming), may benefit social communication and in future studies, infants' and caregivers' rattling should be analyzed in the broader context of communicative behaviors.

This study was focused on how infants produce rattling-movements during play with their caregivers. Our set-up was aimed to give participating infant-mother dyads much freedom and to allow them to play in their preferred way. Therefore, we acknowledge several limitations resulting from this trade-off between a more naturalistic play set-up and controlled conditions. We have not controlled infant posture, and our results include rattling episodes produced across many varied body positions (e.g., lying prone and supine, sitting with and without support, standing) and even during locomotion (rattling while walking). Future studies should further investigate whether between-arms coherence during rhythmic actions is dependent on body positioning. Furthermore, mothers were instructed to play with their infants as they usually do, which means they displayed many different behaviors that may have affected infants' rattling on several levels. First, on a low level, caregivers differed in the structural support they offered to their infants (e.g., for wobbly sitters) and in the encouragement toward rattle-shaking (e.g., handing in a rattle when the infant could not reach for it independently). On a higher level, mothers differed in the prompts for joint-play (rattling together at the same time) and in the number of social cues such as singing or reciting baby rhymes. Our exploratory results do not show significant differences between infants' rattling alone and infants' rattling with their mothers in the average between-arms coherence, rattling frequency or average duration of a rattling episode. However, it may not be possible to fully isolate infants' spontaneous rattling activity from the early attempts at rhythmic coordination with a partner within our experimental set-up. Thus, future research is necessary to measure between-arms coherence by directly testing the same dyads with differing task instructions (asking parents to rattle simultaneously vs. not rattle at all).

## 5. Conclusions

In conclusion, our current findings characterize the longitudinal changes in infants' rhythmic arm movements during rattle-shaking play with their mothers. Infants are similarly motivated to attempt rattle-shaking across the first year of life, but with age, they make more rattling movements and their frequency of rattling increases. Their left and right arm movements become more coupled during rattle-shaking, as shown by the increase in wavelet coherence. Infants also produced more rattling movements and across more rattling episodes in their own rhythm than while being provided with auditory stimulation of mothers' rattling or singing. There were no differences between infants' rattling alone and infants' rattling with their mothers in the average between-arms coherence, rattling frequency or average duration of rattling episode. Our results might shed more light on how spontaneous rhythmic movements can act as precursors of motor social coordination.

## Data availability statement

The datasets presented in this article are not readily available because they will be available upon request from the corresponding authors following an embargo period from the date of publication to allow for the finalization of the ongoing longitudinal project. The computer code used in this study is openly available in GitHub: https://github.com/Mirandeitor/frontiersRhythmicPaper. Requests to access the datasets should be directed to ptomalski@psych.pan.pl.

## Ethics statement

The studies involving human participants were reviewed and approved by Research Ethics Committee at the Institute of Psychology, Polish Academy of Sciences. Written informed consent to participate in this study was provided by the participants' legal guardian/next of kin. Written informed consent was obtained from the individual(s), and minor(s)' legal guardian/next of kin, for the publication of any potentially identifiable images or data included in this article.

## Author contributions

KB, AM-K, ZL, AR, and AK collected data. ZL conducted behavioral coding and performed statistical analyses. AK double-coded 20% of videos. DL performed sensor data pre-processing and wavelet coherence analysis. ZL and DL wrote the first draft of the manuscript. PT acquired funding, contributed main theoretical framework, designed and supervised the overall longitudinal study. All authors contributed to conception and design of the study. All authors contributed to manuscript revision, read, and approved the submitted version.

## Funding

The data collection and analyses, as well as the APC were funded by the Polish National Science Centre grant number 2018/30/E/HS6/00214 to PT.

## Conflict of interest

The authors declare that the research was conducted in the absence of any commercial or financial relationships that could be construed as a potential conflict of interest.

## Publisher's note

All claims expressed in this article are solely those of the authors and do not necessarily represent those of their affiliated organizations, or those of the publisher, the editors and the reviewers. Any product that may be evaluated in this article, or claim that may be made by its manufacturer, is not guaranteed or endorsed by the publisher.
