# Supplementary material for: Longitudinal changes in infants' rhythmic arm movements during rattle-shaking play with mothers
Source: Front Psychol. 2022 Oct 21;13:896319. doi: 10.3389/fpsyg.2022.896319 (PMC9634176; doi:10.3389/fpsyg.2022.896319)
Supplement: Supplementary file 1 [file Data_Sheet_1.pdf]

Supplementary Material

1 S1. CONTROL ANALYSIS 1

For the control purposes, we repeated the analyses excluding infants who had the lowest numbers of rattling episodes (7 rattling episodes or less) to see whether the infrequent rattlers affected the pattern of results. The significance of all main effects remained unchanged apart from the effect of age on the number of rattling episodes (not significant in the control analysis). Furthermore, the difference between observed and shuffled data at T1 was not significant in the control analysis with excluded infrequent rattlers.

1.1 Number of rattling episodes and the average duration of the episode

The number of rattling episodes (annotated periods when infant was holding at least one rattle and made at least one movement that produced rattling noise) did not change with age (Wald $\chi^2(3) = 3.657$, $p = .301$). However, there was a main effect of age in the analysis of the average duration of a rattling episode (Wald $\chi^2(3) = 52.094$, $p < .001$). The duration was shorter at T1 than at T2, T3 and T4 (all ps $< .001$).

1.2 Number of rattling movements and rattling frequency

We predicted that infants would be able to produce more rhythmic hand movements with age. To test this hypothesis, we took the number of hand movements detected automatically in the movement time series during annotated rattling episodes. The number of rattling movements increased with infants' age (Wald $\chi^2(3) = 122.917$, $p < .001$), and pairwise comparisons showed that there were significantly fewer rattling movements at T1 than at T2, T3 and T4 (all ps $< .001$); and fewer at T2 than at T4 ($p = .046$). Furthermore, to calculate the frequency of rattling, we divided the number of rattling movements by the total duration of rattling time. The rattling frequency increased with infants' age (Wald $\chi^2(3) = 21.946$, $p < .001$) and it was lower at T1 than T3 ($p = .029$) and T4 ($p < .001$) and lower at T2 than T4 ($p < .001$).

1.3 Between-hands coherence

The average wavelet coherence increased with age (Wald $\chi^2(3) = 33.974$, $p < .001$) and was significantly larger at T4 than at T1 ($p < .001$), T2 ($p < .001$) and T3 ($p = .003$); longer at T3 than T2 ($p = .034$).

1.4 Control comparisons with shuffled time series

Comparisons of wavelet coherence with the shuffled version showed that at T1 and T2 coordination between both hands is not different from noise (T1: $t(14) = 2.007$; $p = .064$; T2: $t(18) = 0.982$; $p = .339$). For T3 and T4 the coherence for observed data was significantly higher than their corresponding shuffled data (T3: $t(28) = 4.936$; $p < .001$; T4: $t(18) = 7.147$; $p < .001$).

Table S1. *Control Analysis 1: Descriptive statistics at each time point.*

	T1			T2			T3			T4		
	**Mean (SD)**	**Min**	**Max**	**Mean (SD)**	**Min**	**Max**	**Mean (SD)**	**Min**	**Max**	**Mean (SD)**	**Min**	**Max**
**Number of rattling episodes during play**	14.93 (6.37)	8.00	27.00	18.16 (6.40)	8.00	30.00	18.34 (7.12)	8.00	33.00	17.58	9.00	30.00
**Mean duration of rattling episode [s]**	1.52 (0.68)	0.73	3.30	2.68 (1.12)	0.85	6.03	2.97 (0.97)	1.13	5.52	2.92 (1.33)	1.33	5.59
**Number of rattling movements during**	23.80 (9.57)	5.00	39.00	55.11 (33.40)	13.00	141.00	72.31 (27.43)	28.00	124.00	87.11 (43.43)	33.00	195.00
**Rattling frequency [Hz]**	0.96 (0.25)	0.49	1.36	1.04 (0.23)	0.55	1.41	1.22 (0.30)	0.68	1.67	1.42 (0.34)	0.95	2.18
**Wavelet coherence**	0.38 (0.10)	0.23	0.54	0.33 (0.10)	0.16	0.58	0.42 (0.13)	0.19	0.79	0.56 (0.15)	0.37	0.78

2 S2. ANALYSIS OF MOVEMENTS OF A SINGLE HAND: POWER OF WAVELET SPECTRA

To investigate developmental changes in movements of a single hand we calculated the continuous wavelet transform spectra using cwt Matlab function. Power was highest in the frequency range between 2 and 3 Hz consistently at all time points. For further analysis, the power spectra around 1 and 3 Hz were averaged to estimate the power across those frequency ranges. The power of wavelet spectra increased with age (Wald $\chi^2(3) = 18.021$, $p < .001$) as it was higher at T4 than T1 ($p = .001$), T2 ($p < .001$) and T3 ($p = .002$). Our findings suggest that across the first year of life it is not the frequency of rattling that changes, but the organization of rhythmicity within the same frequency range.

Table S2. *Power of wavelet spectra: Descriptive statistics at each time point.*

	T1			T2			T3			T4		
	**Mean (SD)**	**Min**	**Max**	**Mean (SD)**	**Min**	**Max**	**Mean (SD)**	**Min**	**Max**	**Mean (SD)**	**Min**	**Max**
**Power of wavelet spectra**	0.23 (0.18)	0.05	0.97	0.24 (0.17)	0.01	0.77	0.29 (0.16)	0.05	0.72	0.51 (0.30)	0.11	1.25

3 S3. CONTROL ANALYSIS 2

To further analyse infants rattling movements and compare them with results of previous studies, we have re-coded our video data to include only those rattling episodes during which infants consecutively

performed at least 4 arm movements in a row that produced a rattling sound. In addition to infants excluded from the main analysis, we have excluded further 6 infants at T1 and 2 infants at T2 who did not perform 4 rattling movements in a row during a given visit.

3.1 Number of rattling episodes and the average duration of an episode

The number of rattling episodes (annotated periods when an infant was holding at least one rattle and made at least four movements that produced rattling noise) slightly increased with age (Wald $\chi^2(3)$ = 122.814, $p < .001$) as the number of episodes increased between T1 and T2 (p = 0.006), T3 ($p < .001$) and T4 ($p < .001$) as well as between T2 and T4 (p = 0.027). There was no main effect of age in the analysis of the average duration of a rattling episode (Wald $\chi^2(3)$ = 2.922, p = .404).

3.2 Number of rattling movements

The number of rattling movements increased with infants' age (Wald $\chi^2(3)$ = 183.212, $p < .001$), and pairwise comparisons showed that there were significantly fewer rattling movements at T1 than at T2 (p = .003), T3 ($p < .001$) and T4 ($p < .001$); and fewer at T2 than at T3 (p = .009) and T4 (p = .001). The difference between T3 and T4 was not significant (p = .225).

3.3 Rattling frequency

The rattling frequency (i.e. number of rattling movements divided by the total duration of rattling time) increased with infants' age (Wald $\chi^2(3)$ = 11.902, p = .008) and it was higher at T4 than at T1 (p = .019), T2 (p = .010) and T3 (p = .031).

3.4 Between-arms coherence

Average wavelet coherence increased with age (Wald $\chi^2(3)$ = 55.347, $p < .001$) between T2 and T3 ($p < .001$) and between T3 and T4 (p = .033). It was higher at T4 than at T1 (p = .001) or T2 ($p < .001$). The difference between T1 and T2 was not significant (p = .333), similarly there was no difference between T1 and T3 (p = .237).

3.5 Power of wavelet spectra

The power of wavelet spectra increased with infants' age (Wald $\chi^2(3)$ = 13.556, p = .004) and it was higher at T4 than at T1 (p = .024), T2 (p = .002) and T3 (p = .004).

Table S3. *Control Analysis 2: Descriptive statistics at each time point.*

	T1			T2			T3			T4		
	**Mean (SD)**	**Min**	**Max**	**Mean (SD)**	**Min**	**Max**	**Mean (SD)**	**Min**	**Max**	**Mean (SD)**	**Min**	**Max**
**Number of rattling episodes during play**	3.94 (1.89)	1.00	8.00	8.55 (7.35)	1.00	30.00	12.11 (6.63)	2.00	31.00	14.80 (6.44)	2.00	28.00
**Mean duration of rattling episode [s]**	3.59 (1.58)	1.74	6.85	3.99 (1.64)	1.70	10.48	4.19 (1.44)	2.09	8.83	3.89 (1.23)	2.35	6.72
**Number of rattling movements during**	15.22 (7.19)	5.00	31.00	35.23 (31.82)	3.00	127.00	60.00 (30.17)	6.00	118.00	84.15 (45.55)	17.00	194.00
**Rattling frequency [Hz]**	1.21 (0.31)	0.65	1.69	1.20 (0.42)	0.51	2.73	1.29 (0.37)	0.66	2.27	1.55 (0.39)	1.01	2.46
**Wavelet coherence**	0.39 (0.13)	0.23	0.63	0.32 (0.12)	0.14	0.64	0.46 (0.13)	0.28	0.81	0.59 (0.18)	0.22	0.86
**Power of wavelet spectra**	0.28 (0.27)	0.05	1.22	0.28 (0.18)	0.01	0.86	0.30 (0.18)	0.05	0.76	0.55 (0.33)	0.12	1.45

4 S4. ALIGNMENT OF TIME SERIES

The process of alignment of sensor and manually coded data is shown in Supplementary Figures 1-3. First, the period in which clapping was happening in the time series needs to be identified (Figure S1) to correct the existing delay between video and sensor data (Figure S2). Second, after applying *crqad* both time series should be aligned for further processing of the data (Figure S3).

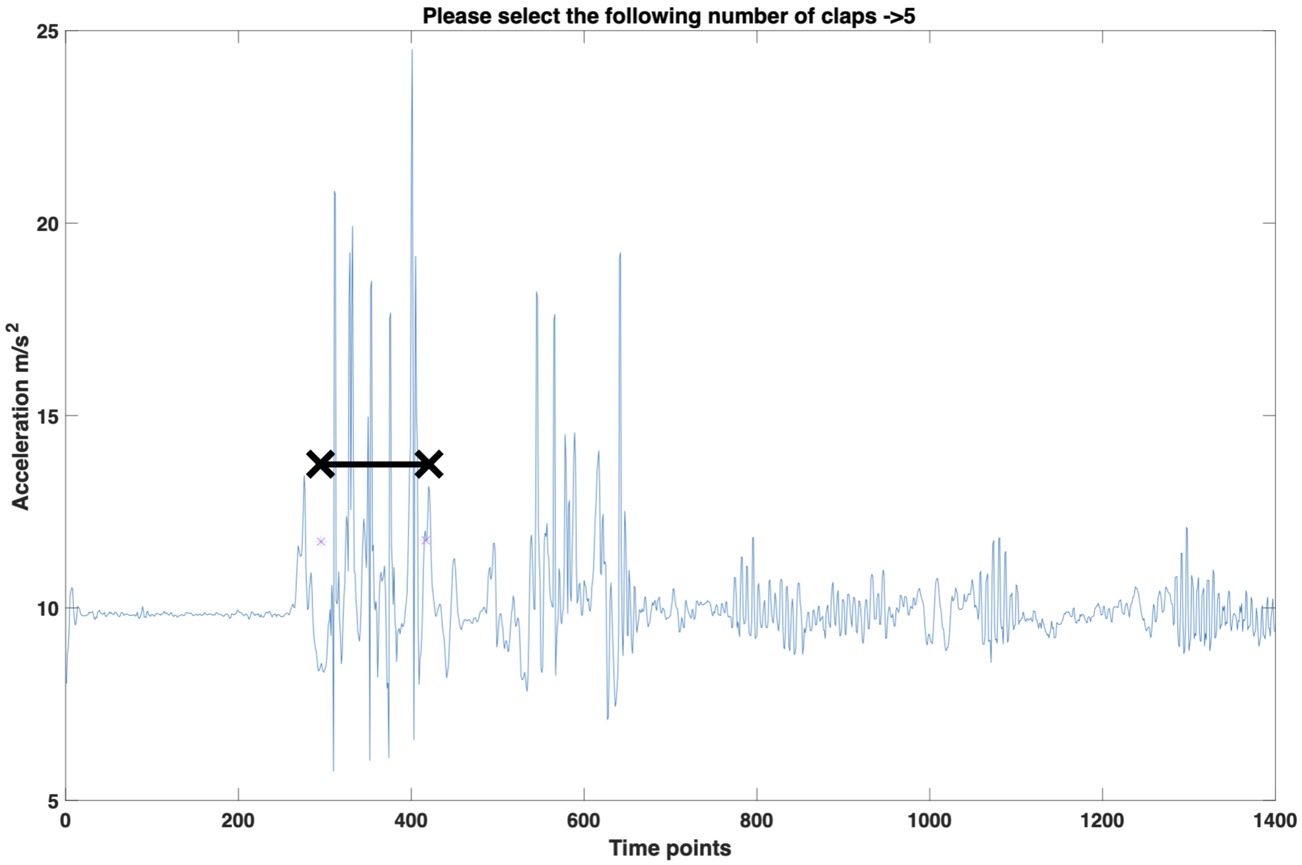

Figure S1. Example of the clapping time series (blue). The title indicates the number of claps that the parent did in that session, which need to be found in the time series. Between black crosses, the identified period where the clapping occurred is marked.

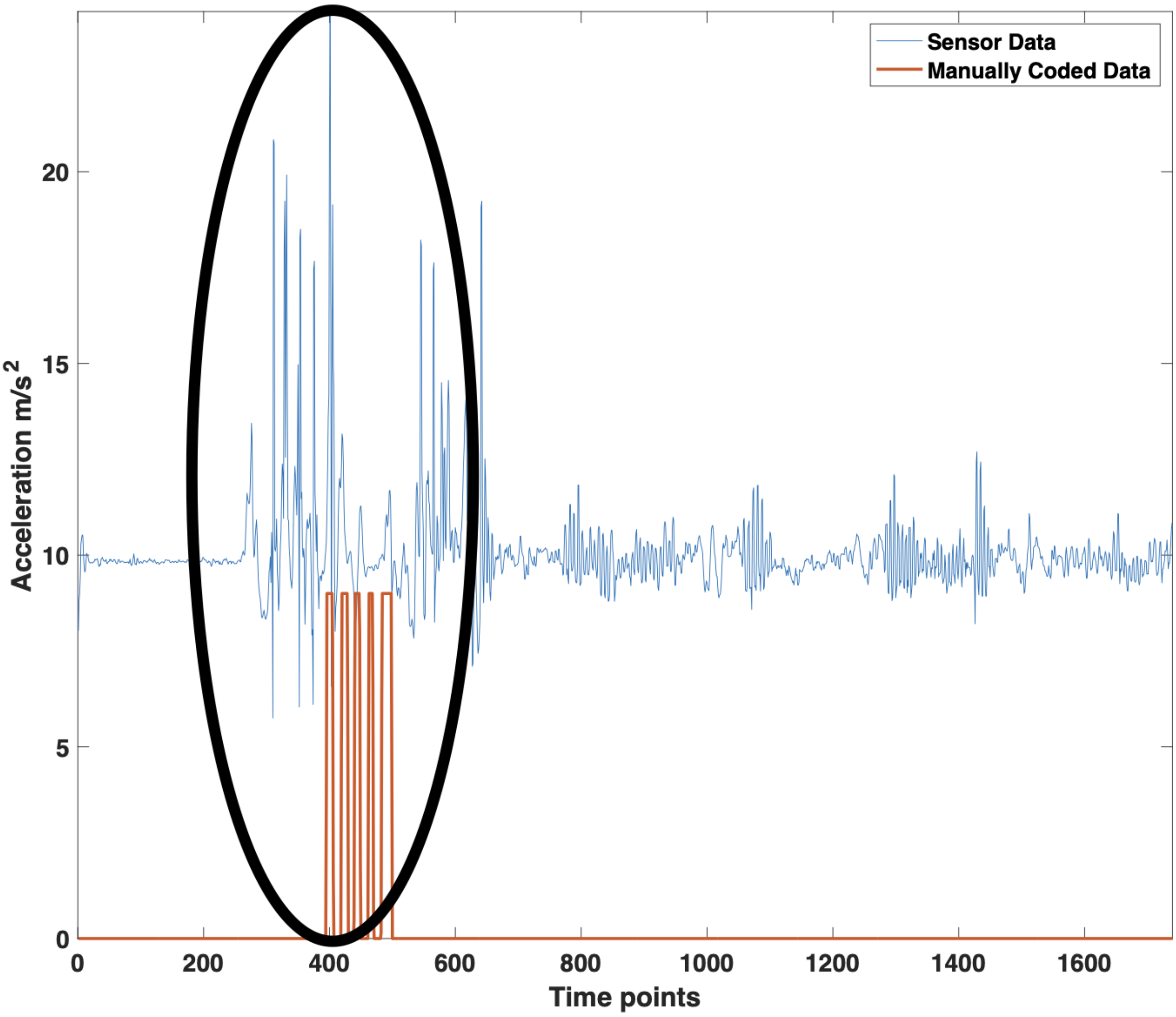

Figure S2. Example of the clapping time series (blue) and the manually coded time series (red). Marked with a black circle is the delay that needs to be corrected between video and sensor data.

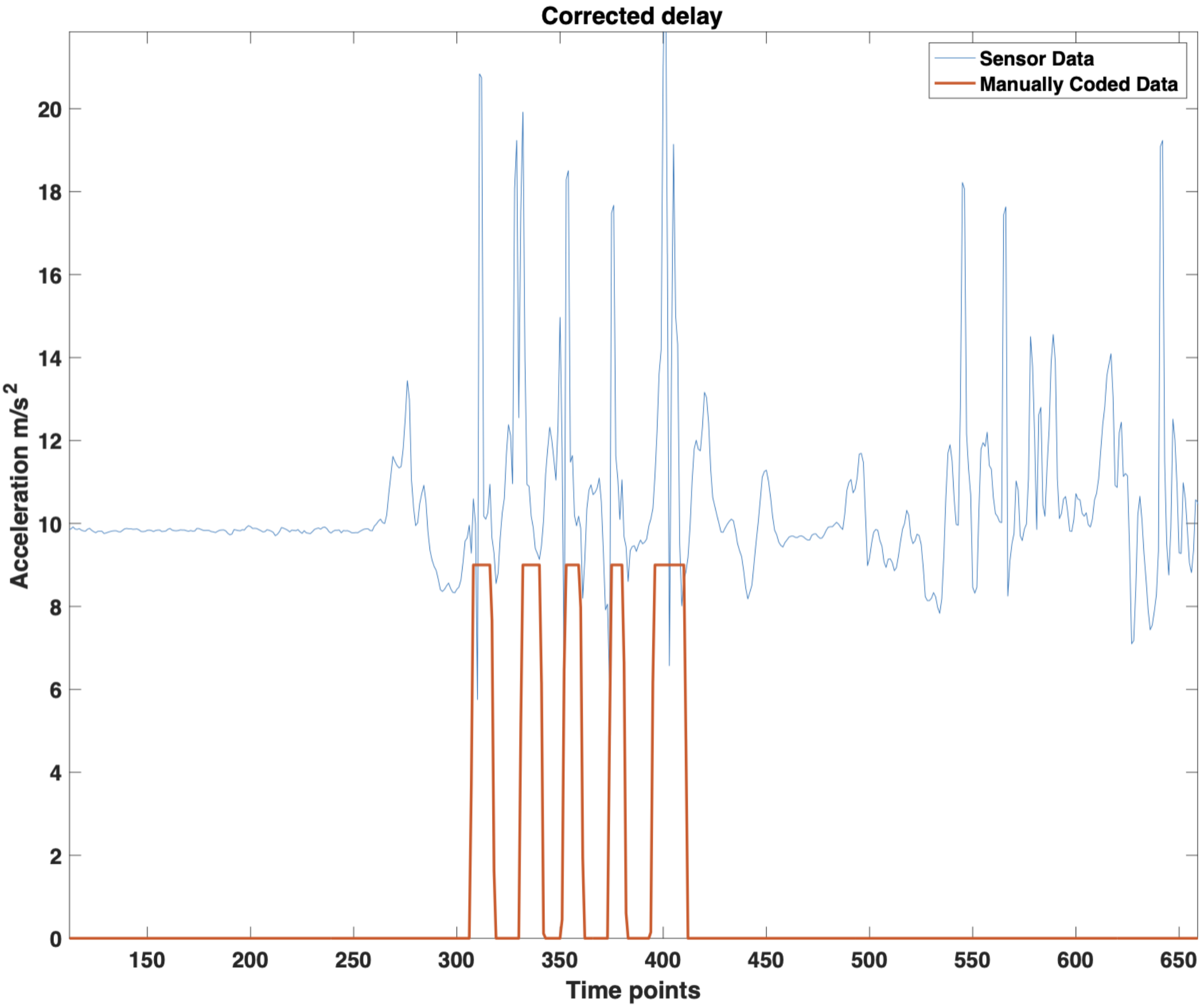

Figure S3. Supplementary Figure 3. Example of the clapping time series (blue) and the corrected manually coded time series (red) after *crqad* analysis has been applied.
